# Effects of a Safety User Interface Bundle on Verification Intentions in Generative AI Chat Use Among Older Chinese Adults: Randomized Vignette Survey

**DOI:** 10.2196/94140

**Published:** 2026-08-14

**Authors:** Jun'an Yu, Jun Chen, Anjie Ren, Hui Duan, Hua Meng, Zhuo Gao

**Affiliations:** 1 Faculty of Science, University of Auckland, Auckland, New Zealand; 2 Medical Services Management Department, Peking University People’s Hospital (PKUPH), Beijing, China; 3 Healthcare & Education Research Center, Chengdu Gongyun Education & Management Research Institute, Chengdu, China; 4 School of Public Administration and Policy, Renmin University of China, Beijing, China; 5 Department of Economics and Management, Sichuan University of Architectural Technology, Chengdu, China; 6 Department of Human Resource Management, Beijing Geriatric Hospital, 118 Wenquan Road, Haidian District, Beijing, 100095, China, 86 15201400966

**Keywords:** generative AI, large language models, user interface, safety cues, verification, trust calibration, older adults, China, vignette survey, randomized experiment, human-computer interaction

## Abstract

**Background:**

Generative AI chat systems are increasingly used for everyday information seeking, but plausible errors and omissions can mislead users when outputs are accepted without scrutiny. Interface-level safety cues may help users calibrate trust and engage in verification; yet, evidence in older Chinese adults remains limited.

**Objective:**

This study aimed to test whether adding a safety user interface (UI) bundle to a generative AI chat interface increases verification intention among older Chinese adults and to examine selected secondary outcomes, including reliance intention, trust calibration, perceived trustworthiness, comprehension, usability/readability, cognitive load, and a behavioral proxy of verification.

**Methods:**

We conducted a cross-sectional survey with an embedded randomized UI vignette experiment between May 22, 2025, and September 3, 2025. Chinese adults aged ≥60 years were recruited through community sites, outpatient clinic waiting areas, and WeChat (Tencent Holdings Ltd) groups, and randomized 1:1 to view screenshots of a baseline chat UI or a safety UI bundle containing generic source-label cues, and an uncertainty and verification nudge. Each participant completed 2 scenarios (service/travel decision and general well-being related to sleep/fatigue), followed by measures of verification intention (primary), reliance intention, trust calibration index, comprehension (0‐8), perceived trustworthiness, usability/readability, cognitive load (0‐10), manipulation checks, and a behavioral proxy (expanding optional “source information”). Analyses used intention-to-treat regression models with covariate adjustment.

**Results:**

Of 214 consenting respondents who started the survey, 200 were included in the analysis (100 per arm). The safety UI bundle increased verification intention (mean 4.72, SD 0.63 vs 4.41, SD 0.59 on a 7-point scale; adjusted β=0.293, 95% CI 0.128-0.457; *P*<.001). Reliance intention did not increase (mean 4.97, SD 0.54 vs 5.03, SD 0.58; adjusted β=−0.105, 95% CI −0.239 to 0.029; *P*=.13). Trust calibration improved (trust calibration index: mean −0.29, SD 1.43 vs 0.29, SD 1.43; adjusted β=−0.567, 95% CI −1.005 to −0.129; P=.01). Expansion of optional source information was numerically higher, although the adjusted CI included the null (42% vs 27%; adjusted odds ratio [OR]=1.76, 95% CI 0.95-3.27; *P*=.07). Comprehension remained high and similar across arms (mean 6.33, SD 1.14 vs 6.32, SD 1.08; adjusted β=−0.132, 95% CI −0.428 to 0.163; *P*=.38). Perceived trustworthiness was modestly lower in the Safety UI arm (mean 5.20, SD 0.61 vs 5.39, SD 0.66; adjusted β=−0.199, 95% CI −0.382 to −0.016; *P*=.03). Usability/readability was unchanged, and cognitive load did not increase. Manipulation checks indicated higher cue recognition in the Safety UI arm.

**Conclusions:**

In a randomized static-vignette survey of older Chinese adults, a brief safety UI bundle was associated with higher verification intention and a trust calibration index consistent with lower overreliance risk, without detectable reductions in comprehension or usability/readability. Because the intervention was tested as a bundle using screenshots and generic source labels, findings should be interpreted as evidence for a practical interface-level strategy rather than proof that any single cue caused the observed effects.

## Introduction

Generative AI chat systems are rapidly becoming a default interface for information seeking and everyday decision support, offering users unprecedented access to synthesized knowledge and conversational assistance across domains such as health, education, and daily living [1-3]. However, the systems are prone to producing plausible but incorrect or incomplete responses, so-called “hallucinations,” which can mislead users if not properly scrutinized [2-4]. The safe use of generative AI thus depends not only on the underlying model’s quality but also on how user interfaces (UIs) shape users’ trust calibration and verification behaviors, especially in high-stakes contexts like digital health or personal decision-making [4,5]. Within human-computer interaction (HCI) and digital health risk frameworks, it is increasingly recognized that interface design plays a critical role in guiding appropriate reliance on AI outputs without overclaiming clinical or societal consequences [2,6].

Older adults represent a particularly high-impact group for generative AI adoption. They stand to benefit substantially from accessible digital tools for health management, communication, and service navigation [7,8]. Yet, older adults may also be more vulnerable to overreliance on automated systems due to lower digital literacy, age-related accessibility constraints, and distinct mental models of technology [9-11]. Research consistently finds that older users face barriers such as small font sizes, complex navigation, and unfamiliar interaction paradigms that can impede effective use or foster misplaced trust [7,8,12]. If UI design can nudge verification behaviors in older adults, encouraging them to check sources or question uncertain outputs, without sacrificing usability or increasing cognitive load, it offers a scalable lever for improving safety in real-world deployments [8,9].

The concept of trust calibration is central to understanding safe human-AI interaction. Trust calibration refers to the alignment between a user’s reliance on an automated system and the system’s actual capabilities and uncertainties [2,4]. Classic literature in automation and HCI demonstrates that people can undertrust (ignoring helpful advice) or overtrust (accepting erroneous recommendations), with both extremes posing risks [4,6]. Interface cues, such as explanations, uncertainty indicators, source attributions, warnings, and verification nudges, are known to influence trust formation and reliance decisions [2,4,13]. Recent work in human-centered AI emphasizes the need for adaptive trust calibration mechanisms that help users match their level of scrutiny to the reliability of the system’s output [4,6].

A growing body of research has explored various “UI safety cues” designed to improve trust calibration in AI and information systems. Such cues include providing explanations for outputs, communicating uncertainty levels, displaying source citations or references, issuing warnings or disclaimers about potential limitations, and designing friction into high-risk decisions [14-19].

Despite the advances, several gaps remain. Most empirical evidence comes from general adult samples in Western contexts or controlled laboratory tasks rather than real-world settings involving older Chinese adults, a population with unique language needs, information ecosystems, and user expectations [10,20]. Few studies have isolated simple UI bundles that are both effective at nudging verification intentions and feasible for deployment in commercial products without retraining underlying models [8,9]. Moreover, much prior work relies heavily on self-reported measures of trust rather than behavioral proxies (eg, actual verification actions), limiting the strength of causal inference about interface effects on safety-related behaviors [3].

This design-evidence gap is particularly salient for design and development teams seeking practical interventions. There is a need for UI changes that can be implemented quickly, without altering model internals, and evaluated efficiently through randomized experiments.

The China context further underscores the importance of targeted research. China has high smartphone penetration among older adults, widespread adoption of messaging platforms (eg, WeChat [Tencent Holdings Ltd]), increasing availability of AI-powered assistants across services, and a rapidly aging population facing unique digital inclusion challenges [10,20]. A China-specific sample enhances external validity by accounting for differences in language processing preferences, local information environments (including censorship or misinformation risks), and culturally shaped expectations about technology authority versus autonomy [10].

This study addresses the gaps by testing whether adding a safety UI bundle—a set of interface cues including uncertainty indicators and citation displays—to a generative AI chat interface increases verification intention among older Chinese adults. The study further examines how this bundle affects related outcomes, including reliance intention (risk of overtrust), perceived trustworthiness of the system, comprehension of content, usability ratings, and subjective cognitive load. Importantly, this work focuses on UI-level interventions rather than model-level performance evaluation.

The anticipated contribution related to older adults was not that all older Chinese adults would necessarily respond in a uniform direction, but that safety cues would be evaluated under conditions common in later-life digital use, including variable digital literacy, high reliance on mobile interfaces, strong need for plain-language guidance, and possible dependence on family or staff assistance. Evidence from younger or more digitally experienced samples may not adequately capture whether low-burden cues remain noticeable, usable, and behaviorally meaningful in this population.

By providing randomized evidence on a practical bundle of interface cues tailored for older Chinese users and by quantifying the trade-off between increased verification orientation versus potential reductions in perceived trustworthiness, the study offers cautious design evidence for developers targeting safer AI use while recognizing that component-level effects require separate testing.

## Methods

### Study Design

We conducted this cross-sectional survey with an embedded randomized UI vignette experiment between May 22, 2025, and September 3, 2025. A 2-arm, between-subjects design compared a baseline generative AI chat interface with a safety UI bundle. Participants were randomized in a 1:1 ratio. Each participant viewed 2 scenarios in the assigned UI condition and completed outcome measures after each scenario. Analyses followed an intention-to-treat approach based on randomized assignment.

### Setting and Participant Recruitment

Participants were Chinese adults aged 60 years or older residing in mainland China. Recruitment was conducted through 3 channels, including community-based recruitment at senior activity centers or neighborhood community sites, outpatient clinic waiting area recruitment with on-site research staff, and online recruitment via WeChat groups that included older adults. To reduce exclusion of individuals with lower digital literacy, family-assisted or interviewer-assisted completion was permitted. Assistance mode was recorded for all participants and was included as a covariate and in sensitivity analyses. The survey was administered in simplified Chinese.

### Eligibility Criteria

Inclusion criteria were age 60 years or older, residence in mainland China, ability to read Chinese or complete the survey with assistance, and provision of informed consent. Exclusion criteria were inability to provide informed consent, duplicate submissions identified using platform controls plus response-pattern review, and low-quality responses defined a priori as failure on an instructed-response attention check combined with an implausibly short completion time.

### Duplicate Detection

For online recruitment, the survey platform settings were used to reduce repeat submissions, and additional screening was performed using completion time, highly similar response patterns, and device or IP-based indicators available to the platform. For community and clinic recruitment, staff recorded whether a participant had already completed the survey on the same day, and responses were additionally screened post hoc for near-identical response patterns and implausible repetition across entries.

### Randomization and Allocation Concealment

Randomization was implemented within the survey platform using built-in equal-probability assignment in a 1:1 ratio. Allocation was concealed until assignment. Condition assignment was maintained across both scenarios for each participant.

### Experimental Conditions and Rationale for UI Choices

The intervention was defined as a practical safety UI bundle rather than separable component-level manipulations. The study was not designed to attribute effects to individual elements within the bundle, and inferences were limited to the bundled condition compared with baseline.

The bundle development process was pragmatic and reproducibility-oriented. The research team first identified interface elements that could be implemented without model retraining or retrieval-system changes, then selected low-salience source labels and a short uncertainty plus verification message that could fit within a mobile chat layout. Candidate wording was simplified to avoid technical terms, preserve readability for older adults, and avoid giving the impression that the generic source labels represented verified citations.

#### Condition 1: Baseline UI

Participants viewed static screenshots of a mobile chat interface displaying one user prompt and one generative AI answer. The interface did not display additional safety cues under the answer. Screenshots of the baseline interface for both scenarios are provided in Figure S1A and S1C in Multimedia Appendix 1.

#### Condition 2: Safety UI Bundle

Participants viewed screenshots of the same interface and the same prompt and answer text, with 2 additional UI elements placed under the answer.

##### Source-Label Cue

A “来源” (source) label followed by 2-3 neutral source chips, defined here as compact pill-shaped labels placed below the AI answer (for example “权威来源A” [authoritative source A], “权威来源B” [authoritative source B], and “公共服务信息来源” [public service information source]). To avoid implying verified provenance, the chips were intentionally generic and were presented as interface labels rather than citations of underlying model retrieval. A brief note indicated that source information could be expanded.

##### Uncertainty and Verification Nudge

A short panel stated that the answer might be incomplete or not suitable for individual circumstances and recommended checking authoritative sources and consulting professionals when appropriate. The wording was drafted by the research team with attention to plain Chinese, low literacy burden, and avoidance of diagnostic or individualized clinical instruction; however, it was not developed through a separate formal linguistic validation process or review by a behavioral health expert panel.

### Vignette Scenarios and Stimulus Construction

Two scenarios were developed to reflect common information needs among older adults while minimizing potential ethical and clinical risks. Scenario 1 addressed an everyday service or travel decision query. Scenario 2 addressed general well-being related to sleep quality and daytime fatigue. The generative AI answers were written conservatively and avoided diagnosis, medication dosing, or individualized clinical instruction.

Stimuli were presented as static mobile UI screenshots with large, high-contrast text. The interface layout, typography, spacing, icons, and answer length were held constant across conditions. Only the safety UI elements differed between arms. Participants could not interact with the chat interface itself, so the experiment captured responses to a controlled representation of an AI chat interface rather than the cognitive demands of real-time conversational use. Screenshots of the safety UI bundle for both scenarios are provided in Figures S1A-S2B and in Multimedia Appendix 1.

### Scenario Order

To reduce order effects, the presentation order of the 2 scenarios was randomized at the participant level by the survey platform. Participants remained in the same assigned UI condition regardless of scenario order.

### Procedure

After informed consent and eligibility screening, participants completed baseline measures including demographics, digital literacy, technology anxiety, and prior exposure to AI chat tools. Participants were then randomized to the baseline UI or safety UI bundle condition. Participants viewed the first scenario screenshot (assigned condition) and completed scenario-specific outcome measures, a brief manipulation check, and a comprehension quiz. Participants then viewed the second scenario screenshot (same assigned condition) and completed the same measures. The survey concluded with brief global attitude items and an optional open-ended question about desired interface features. Completion time and per-page dwell time were recorded automatically. Assistance mode (independent, family-assisted, and interviewer-assisted) was recorded for all participants.

Family-assisted or interviewer-assisted completion was allowed when participants had visual, motor, or digital-literacy barriers. During assisted completion, participants were shown the assigned screenshots whenever possible. If reading support was required, assistants were instructed to read the visible on-screen text and survey questions verbatim, including any source-label text or uncertainty/verification nudge displayed in the assigned condition, without explaining, emphasizing, or interpreting the interface cues or the intended purpose of the intervention. Assistance mode was recorded for all participants.

### Behavioral Proxy Measure of Verification Behavior

To strengthen outcome validity beyond self-report, a behavioral proxy for verification was included after each scenario. Participants were shown a small, nonemphasized expandable element labeled “来源信息 (可展开)” (source information [expandable]) placed below the outcome questions, not directly under the screenshot. Expanding it opened a brief neutral source information panel. The outcome was whether the participant expanded it at least once. This measure was interpreted as a low-friction proxy for curiosity about source information, not as evidence of substantive real-world verification such as cross-checking external websites, consulting clinicians, or comparing multiple information sources.

### Measures

All Likert items were measured on a 7-point scale from “非常不同意” (strongly disagree) to “非常同意” (strongly agree), unless otherwise specified.

### Manipulation Checks

After each scenario, participants completed two brief manipulation check items: (1) a yes/no item assessing whether the interface displayed a source information prompt, and (2) a Likert item assessing whether they noticed “提示类信息” (tip-style information, including reminders to verify or consult professionals).

### Primary Outcomes

Verification intention was the primary outcome of the study. For each scenario, verification intention was measured using a 3-item study-created scale informed by prior work on information verification and AI reliance. The items assessed intention to cross-check information via other channels, preference for authoritative sources, and intention to consult professionals or experienced individuals before acting. Scores were averaged across scenarios and items; higher scores indicated stronger verification intention.

Reliance intention was a co-primary outcome. For each scenario, reliance intention was measured using a 3-item study-created scale informed by automation-trust and AI-adoption literature. The items assessed willingness to follow the advice, perceived sufficiency to adopt the advice directly, and likelihood of using the AI chat interface again for similar problems. A reliance intention score was averaged across scenarios and items; higher scores indicated stronger direct reliance intention.

The behavioral proxy expansion outcome was treated as a co-primary supportive measure. It was defined as expanding “来源信息 (可展开)” at least once, with scenario-level expansion reported as a secondary breakdown.

### Secondary Outcomes

#### Trust Calibration Index

A trust calibration index was computed as an exploratory secondary representation of calibration. It was defined a priori as the standardized difference between reliance intention and verification intention, averaged across scenarios, with higher values indicating greater overreliance risk. Reliance and verification intention were standardized within the analytic sample before subtraction. This index was study-created and was not a previously validated psychometric instrument; it was intended to operationalize the balance between direct reliance and verification orientation in this vignette context.

#### Comprehension

Four study-created multiple-choice questions per scenario assessed understanding of key points stated in the answer, including the main recommendation, stated uncertainty or caveat, appropriate next action, and an item requiring recognition of information that the answer did not provide. Each item was scored as correct or incorrect, yielding a total comprehension score from 0 to 8 across scenarios.

#### Perceived Trustworthiness

A 4-item study-created composite, informed by human-AI trust literature, assessed perceived competence, overall reliability, perceived risk of being misleading (reverse-coded), and perceived helpfulness. Scores were averaged per scenario and across scenarios. Internal consistency was assessed in the analytic sample.

#### Perceived Usability and Readability

A 5-item study-created composite assessed text readability, ease of locating key points, ease of understanding, confidence in using the interface, and overall satisfaction. The composite was not a direct administration of the System Usability Scale because the experiment used static screenshots rather than a fully interactive system. Scores were averaged per scenario and across scenarios, and internal consistency was assessed.

#### Cognitive Load

A single mental-effort item per scenario, adapted from established subjective cognitive load measurement practice, was rated from 0 (no effort) to 10 (very high effort) and assessed perceived effort to read and understand the answer. Scores were averaged across scenarios.

### Determinants and Covariates

Digital literacy was measured using a 6-item study-created screener assessing self-rated ability to perform smartphone-based information search, app installation and updating, font-size adjustment, voice input use, privacy settings management, and handling common login or verification steps. Items were scored on a 5-point scale and averaged, with higher scores indicating greater digital literacy. Participants who completed the survey with family or interviewer assistance were not excluded from digital literacy analyses solely because assistance was provided. Their digital literacy data were retained as participant-reported baseline characteristics. Assistance mode was recorded separately for all participants and was included as a covariate in adjusted models to account for potential differences between independent, family-assisted, and interviewer-assisted completion.

Technology anxiety was measured using a 4-item study-created scale informed by prior technology-anxiety constructs. The items assessed nervousness with new technology, worry about making mistakes, avoidance of unfamiliar features, and perceived stress when learning technology. Items were averaged, with higher scores indicating greater technology anxiety.

Covariates included age, gender, education level, residence type, living arrangement, self-rated health, prior AI chat exposure, and assistance mode.

### Data Quality Procedures

The survey included an instructed-response attention check. Duplicate prevention used survey platform settings plus post hoc response-pattern checks. Dwell time and completion time were recorded and used as quality indicators rather than stand-alone enforced thresholds to avoid disproportionate burden on older adults who may need more time or assistance. Low-quality responding was defined before analysis as failure of the instructed-response attention check plus either an implausibly short total completion time (<3 minutes) or a straight-line response pattern across all Likert outcome items.

### Sample Size

The target sample size was set at 200 participants (approximately 100 per arm). This sample size was selected to support estimation of the between-group difference in the primary continuous outcome (verification intention) with adequate precision while remaining feasible across the recruitment channels. Using a 2-sample comparison framework with equal allocation, 2-sided α of .05, and a continuous outcome, a total sample of 200 provided approximately 80% power to detect a standardized mean difference of about 0.4 between arms. The sample size also supported adjusted regression models including prespecified covariates without unstable estimation. The study was powered for the primary main effect comparison; heterogeneity analyses were interpreted descriptively.

### Statistical Analysis

All analyses were conducted under intention-to-treat based on randomized assignment. Baseline balance between arms was described using standardized mean differences for key demographic and technology-related variables.

Primary analyses estimated the effect of condition (safety UI bundle vs baseline) on verification intention and reliance intention using linear regression with robust SEs, adjusting for prespecified covariates (age, gender, education, prior AI chat exposure, digital literacy, technology anxiety, and assistance mode). Results were reported as adjusted mean differences with 95% CIs. For verification intention, an unadjusted Cohen *d* was also reported descriptively.

The behavioral proxy expansion outcome was analyzed using logistic regression estimating the odds of expanding “来源信息 (可展开)” at least once, adjusting for the same covariates. Scenario-level expansion outcomes were summarized descriptively and analyzed in secondary models.

Secondary outcomes, including trust calibration index, perceived trustworthiness, usability/readability, and cognitive load, were analyzed using analogous regression models. For the comprehension score, distributional diagnostics were examined to determine whether a count model was necessary; because diagnostics did not indicate material model misspecification, the final analysis used linear regression with robust SEs, and effects were reported as adjusted mean differences on the 0-8 score scale.

Because assistance during completion could influence participants’ ability to complete the survey and could be associated with digital literacy, assistance mode was adjusted for in the primary models. In addition, a sensitivity analysis restricted to participants who completed the survey independently was conducted to assess whether the main findings were robust to exclusion of assisted completions.

A condition-by-digital literacy interaction term was examined to assess whether the intervention effect varied with digital literacy, and results were interpreted alongside uncertainty intervals.

### Missing Data Handling

Item-level missingness was below the predefined 5% threshold for all primary and secondary outcomes; therefore, complete-case analysis was used for the final analysis. Multiple imputation by chained equations was not applied.

### Ethical Considerations

Ethics approval was obtained from the Ethics Committee of Chengdu Gongyun Education and Management Research Institute (approval number GYLL25013). Although this study uses a randomized allocation design to present different user interface variants, it is classified as a cross-sectional vignette survey rather than a clinical randomized controlled trial involving a live intervention, and therefore clinical trial database registration was not required. Because the study involved survey responses and static vignettes rather than clinical intervention, it was classified as minimal risk. All participants provided informed consent before eligibility screening and randomization. Study data were deidentified before analysis, and no identifying clinical information was collected. No participant images or identifiable user screenshots were included in the manuscript or supplementary materials. Participants did not receive monetary compensation. The well-being scenario provided general nondiagnostic information only and advised professional consultation when appropriate. Family-assisted or interviewer-assisted completion was permitted for accessibility; research staff were not involved in outcome interpretation, but on-site staff were not formally blinded to randomized condition after assignment because screenshots were visible during assisted completion.

## Results

### Internal Consistency and Descriptive Performance of Study-Created Measures

Item-level missingness was 0% for the study-created multi-item measures used in the primary and covariate-adjusted analyses. Internal consistency was good to excellent for the 2 primary self-report outcomes. Cronbach α was 0.890 for the 6-item verification intention score and 0.873 for the 6-item reliance intention score. Scenario-specific coefficients were also acceptable for verification intention and reliance intention, as shown in Table 1.

**Table 1.** Internal consistency and scoring of study-created multi-item measures.

Measure^a^	Items	Range	Score construction	Cronbach α	Scenario 1/2 (Cronbach α)	Overall mean (SD)
Verification intention	6	1‐7	Mean of 3 items in each of 2 scenarios	0.890	0.829/0.785	4.57 (0.63)
Reliance intention	6	1‐7	Mean of 3 items in each of 2 scenarios	0.873	0.770/0.792	5.00 (0.56)
Digital literacy	6	1‐5	Mean of 6 baseline screener items	0.959	—^b^	3.14 (0.78)
Technology anxiety	4	1‐7	Mean of 4 baseline anxiety items	0.941	—	3.66 (1.27)
Perceived trustworthiness	8	1‐7	Mean of 4 items in each of 2 scenarios	0.930	0.855/0.881	5.29 (0.66)
Usability/readability	10	1‐7	Mean of 5 items in each of 2 scenarios	0.946	0.898/0.901	5.40 (0.63)

a Verification intention, reliance intention, perceived trustworthiness, and usability/readability were measured after each of the 2 vignette scenarios and then averaged at the participant level. Digital literacy and technology anxiety were baseline covariate scales.

b Not applicable.

The study-created covariate scales also showed high internal consistency. Cronbach α was 0.959 for the 6-item digital literacy screener and 0.941 for the 4-item technology anxiety scale. For the secondary composite outcomes, α was 0.930 for perceived trustworthiness and 0.946 for usability/readability. The scoring rules, overall descriptive statistics, and reliability coefficients for the study-created multi-item measures are summarized in Table 1.

The descriptive distributions were consistent with the primary outcome pattern and did not indicate problematic floor or ceiling effects for the main self-report scales. Verification intention was higher in the Safety UI bundle arm, whereas reliance intention was similar between arms. Digital literacy and technology anxiety were also similar between randomized arms, supporting their role as covariates rather than major sources of baseline imbalance. Descriptive performance for the study-created scales and nonscale measures is reported in Table 2.

**Table 2.** Descriptive performance of study-created measures by randomized arm.

Measure	Range	Baseline UI^a^ (n=100)	Safety UI bundle (n=100)	Interpretation
Verification intention, mean (SD)	1‐7	4.41 (0.59)	4.72 (0.63)	Higher scores indicate stronger intention to verify.
Reliance intention, mean (SD)	1‐7	5.03 (0.58)	4.97 (0.54)	Higher scores indicate stronger direct reliance.
Digital literacy, mean (SD)	1‐5	3.12 (0.78)	3.15 (0.78)	Baseline covariate; higher scores indicate greater digital literacy.
Technology anxiety, mean (SD)	1‐7	3.66 (1.29)	3.67 (1.25)	Baseline covariate; higher scores indicate greater technology anxiety.
Perceived trustworthiness, mean (SD)	1‐7	5.39 (0.70)	5.20 (0.61)	Higher scores indicate greater perceived trustworthiness.
Usability/readability, mean (SD)	1‐7	5.40 (0.60)	5.39 (0.66)	Higher scores indicate better usability/readability.
Comprehension total, mean (SD)	0‐8	6.32 (1.08)	6.33 (1.14)	Objective quiz total; alpha was not interpreted.
Cognitive load, mean (SD)	0‐10	3.96 (1.60)	3.56 (1.44)	Mean of one mental-effort item per scenario.
Expanded source information at least once, n (%)	0/1	27 (27.0)	42 (42.0)	Behavioral proxy; not a psychometric scale.

a UI: user interface.

The comprehension outcome was scored as an 8-item objective quiz total and was not treated as a reflective psychometric scale because the items intentionally sampled distinct factual and caveat-related elements from the 2 vignettes. Cognitive load was assessed using one mental-effort rating per scenario, and source-information expansion was a binary behavioral proxy; therefore, Cronbach α was not interpreted for these measures.

Overall, the reliability and descriptive results support the internal consistency and scoring adequacy of the study-created verification intention, reliance intention, digital literacy, technology anxiety, perceived trustworthiness, and usability/readability scores. These findings provide measurement support for the adjusted intention-to-treat analyses of the primary and secondary outcomes.

### Participant Flow and Analytic Sample

**Figure 1.**
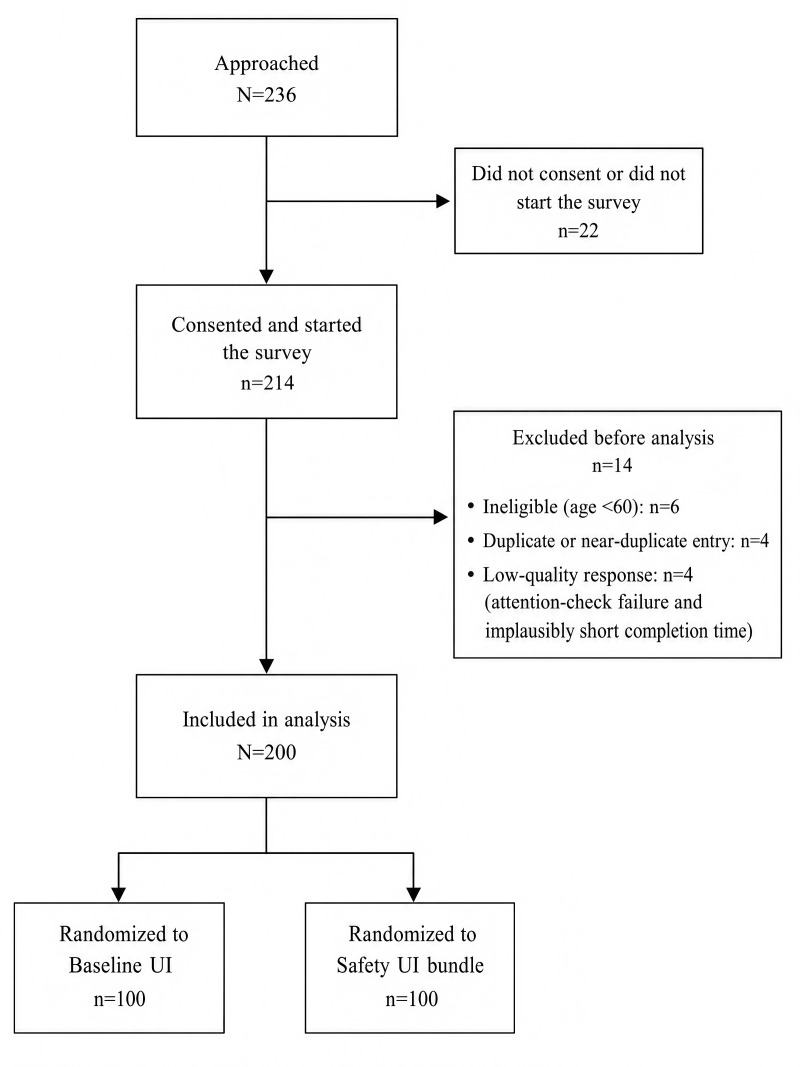
Participant flow diagram for a randomized vignette survey of a safety user interface (UI) bundle for generative AI chat use among older Chinese adults in mainland China, May 22, 2025-September 3, 2025. The diagram shows the numbers approached, not consenting or not starting the survey, consenting and starting the survey, excluded before analysis, and included in each randomized arm, with reasons for exclusion.

A total of 236 individuals were approached across the 3 recruitment channels. Twenty-two individuals did not consent or did not start the survey. Of 214 who provided consent and started the survey, 14 were excluded before analysis because of ineligibility (n=6, <60 years), duplicate or near-duplicate entries (n=4), or low-quality responding defined as failing the attention check with an implausibly short completion time (n=4). The final analytic sample included 200 participants, with 100 randomized to the Baseline UI arm and 100 randomized to the Safety UI bundle arm (Figure 1).

### Baseline Characteristics

Randomization produced broadly comparable arms (Table 3), although some baseline differences were observed. The mean age was 67.48 (SD 5.14) years in the Baseline UI arm and 67.65 (SD 5.18) years in the Safety UI arm. Female participants accounted for 54.0% (n=54) in the Baseline UI arm and 44.0% (n=44) in the Safety UI arm. College or higher education was reported by 14.0% (n=14) in the Baseline UI arm and 30.0% (n=30) in the Safety UI bundle arm. Prior use of AI chat tools was reported by 40.0% (n=40) in the Baseline UI arm and 41.0% (n=41) in the Safety UI arm. Standardized mean differences were largest for education and gender, so adjusted analyses retained prespecified covariates to reduce residual confounding.

**Table 3.** Baseline demographic, health, technology-use, and survey-completion characteristics of participants (N=200).

Characteristic	Baseline UI^a^ (n=100)	Safety UI bundle (n=100)
Age (years), mean (SD)	67.48 (5.14)	67.65 (5.18)
Sex (female), n (%)	54 (54.0)	44 (44.0)
Education, n (%)		
Primary or less	14 (14.0)	16 (16.0)
Middle school	42 (42.0)	24 (24.0)
High school or vocational	30 (30.0)	30 (30.0)
College or higher	14 (14.0)	30 (30.0)
Residence type, n (%)		
Urban	50 (50.0)	47 (47.0)
County	31 (31.0)	32 (32.0)
Rural	19 (19.0)	21 (21.0)
Assistance mode, n (%)		
Independent	68 (68.0)	66 (66.0)
Family-assisted	17 (17.0)	18 (18.0)
Interviewer-assisted	15 (15.0)	16 (16.0)
AI chat ever used, n (%)	40 (40.0)	41 (41.0)
Digital literacy (1-5), mean (SD)	3.12 (0.78)	3.15 (0.78)
Technology anxiety (1-7), mean (SD)	3.66 (1.29)	3.67 (1.25)
Self-rated health (1-5), mean (SD)	3.46 (0.82)	3.46 (0.90)
Scenario 1 presented first, n (%)	52 (52.0)	52 (52.0)

a UI: user interface.

### Manipulation Checks

The manipulation checks confirmed that participants perceived the intended UI differences. In the Safety UI bundle arm, 75.0% (n=75) reported noticing a source prompt, compared with 15.0% (n=15) in the Baseline UI arm. The “noticed tip” item also differed in the expected direction, with mean 5.20 (SD 1.13) in the Safety UI bundle arm and mean 3.26 (SD 1.15) in the Baseline UI arm (Table 4).

**Table 4.** Manipulation checks after exposure to baseline and safety user interface (UI) bundle screenshots (N=200).

Measure	Baseline UI^a^ (n=100)	Safety UI bundle (n=100)
Noticed source prompt, n (%)	15 (15.0)	75 (75.0)
Noticed tip item (1-7), mean (SD)	3.26 (1.15)	5.20 (1.13)

a UI: user interface.

### Primary Outcomes

Verification intention was higher in the Safety UI bundle arm than in the Baseline UI arm. The mean verification intention score was 4.41 (SD 0.59) in the Baseline UI arm and 4.72 (SD 0.63) in the Safety UI bundle arm, corresponding to an unadjusted mean difference of 0.31 points on the 7-point scale (Cohen *d*=0.50). In covariate-adjusted regression, assignment to the Safety UI bundle was associated with a 0.293 point increase in verification intention (95% CI 0.128-0.457; *P*<.001), holding constant age, gender, education, prior AI chat use, digital literacy, technology anxiety, and assistance mode.

Reliance intention was similar between arms. The mean reliance intention score was 5.03 (SD 0.58) in the Baseline UI arm and 4.97 (SD 0.54) in the Safety UI bundle arm, with a small unadjusted difference of −0.06. In covariate-adjusted regression, the estimated association between the Safety UI bundle and reliance intention was −0.105 (95% CI −0.239 to 0.029; *P*=.13).

The behavioral proxy outcome showed a numerically higher proportion of participants expanding the optional “source information” element in the Safety UI bundle arm, but the adjusted estimate was not statistically definitive. Expansion at least once occurred in 27.0% (n=27) of Baseline UI participants and 42.0% (n=42) of Safety UI participants. In adjusted logistic regression, the odds ratio (OR) for expansion was 1.76 (95% CI 0.947-3.269; *P*=.07), suggesting a possible increase in source-information seeking, although the CI included the null.

Scenario-level expansion proportions were numerically higher in the Safety UI bundle arm, but these secondary binary outcomes were interpreted cautiously. In Scenario 1, expansion occurred in 21.0% (n=21) of participants in the Baseline UI arm and 34.0% (n=34) in the Safety UI bundle arm. In the adjusted logistic regression model controlling for age, gender, education, prior AI chat exposure, digital literacy, technology anxiety, and assistance mode, assignment to the Safety UI bundle was associated with higher odds of Scenario 1 expansion, although the CI included the null (adjusted OR=1.86, 95% CI 0.94-3.68; *P*=.08). In Scenario 2, expansion occurred in 18.0% (n=18) of participants in the Baseline UI arm and 31.0% (n=31) in the Safety UI bundle arm. The adjusted scenario-level model similarly favored the Safety UI bundle, again with uncertainty around the estimate (adjusted OR=1.92, 95% CI 0.95-3.88; *P*=.07). These scenario-level models were interpreted as secondary supportive analyses because the study was powered for the main continuous outcome rather than for scenario-specific binary expansion outcomes. Global attitude items were exploratory and showed no evidence that the safety UI bundle reduced willingness to use AI chat tools for low-risk everyday information seeking. Optional open-ended responses were sparse and were not treated as formal outcomes (Table 5).

**Table 5.** Primary verification, reliance, and behavioral proxy outcomes by randomized arm (N=200).

Outcome	Baseline UI^a^ (n=100)	Safety UI bundle (n=100)	Unadjusted difference^b^	Adjusted effect estimate^c^, estimate (95% CI)	*P* value
Verification intention (1-7), mean (SD)	4.41 (0.59)	4.72 (0.63)	0.31	β=0.293 (0.128 to 0.457)	<.001
Reliance intention (1-7), mean (SD)	5.03 (0.58)	4.97 (0.54)	–0.06	β=–0.105 (–0.239 to 0.029)	.13
Expanded source info at least once, n (%)	27 (27.0)	42 (42.0)	15.0	OR^d^ 1.76 (0.95 to 3.27)	.07

a UI: user interface.

b Unadjusted difference was calculated as the Safety UI bundle arm minus the Baseline UI arm: mean difference for continuous outcomes and percentage point difference for the binary behavioral proxy.

c Adjusted estimates controlled for age, gender, education, prior AI chat exposure, digital literacy, technology anxiety, and assistance mode.

d OR: odds ratio.

### Secondary Outcomes

Trust calibration, operationalized as the standardized difference between reliance and verification, favored the Safety UI bundle arm. The trust calibration index mean was 0.29 (SD 1.29) in the Baseline UI arm and −0.29 (SD 1.43) in the Safety UI bundle arm, indicating lower overreliance risk when the safety UI bundle was present. The adjusted association also favored the Safety UI bundle (β=−0.567, 95% CI −1.005 to −0.129; *P*=.01). Because the generative AI answers were intentionally conservative and did not include incorrect or hallucinated responses, the index should be interpreted as an exploratory balance between reliance and verification orientation rather than a definitive measure of correct trust calibration.

Comprehension scores were high in both arms and nearly identical. The mean comprehension total score was 6.32 (SD 1.08) in the Baseline UI arm and 6.33 (SD 1.14) in the Safety UI bundle arm. Distributional diagnostics did not indicate a need for a count model; therefore, the final adjusted analysis used linear regression with robust SEs. The adjusted association between arm and comprehension was small and not statistically distinguishable from zero (β=−0.132, 95% CI −0.428 to 0.163; *P*=.38).

Before outcome modeling, internal consistency was assessed for the 2 study-created multi-item secondary outcome composites. Cronbach α was 0.930 for perceived trustworthiness and 0.946 for usability/readability, supporting use of the averaged composite scores in the regression analyses.

Perceived trustworthiness showed a modest reduction in the Safety UI bundle arm. The mean perceived trustworthiness score was 5.39 (SD 0.70) in the Baseline UI arm and 5.20 (SD 0.61) in the Safety UI bundle arm. The adjusted β was −0.199 (95% CI −0.382 to −0.016; *P*=.03), indicating that adding safety cues improved verification orientation while slightly lowering perceived trustworthiness.

**Figure 2.**
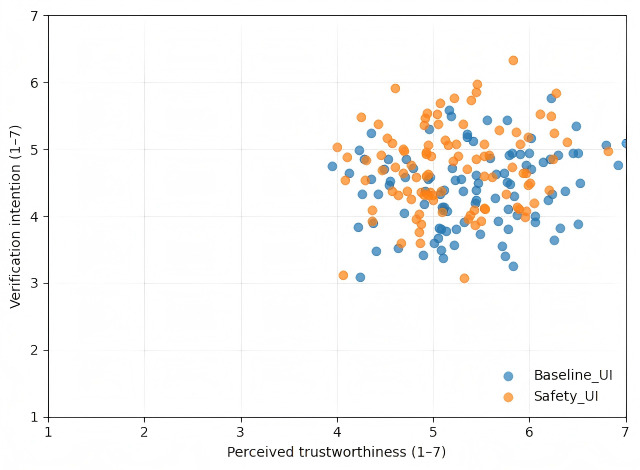
Trade-off between verification intention and perceived trustworthiness by randomized arm in a static generative AI chat vignette survey among older Chinese adults in mainland China, May 22, 2025-September 3, 2025. The scatter plot displays participant-level verification intention and perceived trustworthiness scores, with color indicating the randomized arm. UI: user interface.

Usability/readability ratings were similar between arms, with mean 5.40 (SD 0.60) in the Baseline UI arm and 5.39 (SD 0.66) in the Safety UI bundle arm. The adjusted association was small and not statistically significant (β=−0.069, 95% CI −0.254 to 0.115; *P*=.46). Cognitive load tended to be lower in the Safety UI bundle arm, with mean 3.96 (SD 1.60) in the Baseline UI arm and 3.56 (SD 1.44) in the Safety UI bundle arm, although the adjusted estimate did not exclude zero (β=−0.346, 95% CI −0.762 to 0.071; *P*=.10; Figure 2 and Table 6).

**Table 6.** Secondary trust calibration, comprehension, perceived trustworthiness, usability/readability, and cognitive load outcomes by randomized arm (N=200).

Outcome	Baseline UI^a^ (n=100)	Safety UI bundle (n=100)	Unadjusted difference^b^	Adjusted effect estimate^c^, β (95% CI)	*P* value
Trust calibration index, mean (SD)	0.29 (1.29)	–0.29 (1.43)	–0.58	–0.567 (–1.005 to −0.129)	.01
Comprehension total (0-8), mean (SD)	6.32 (1.08)	6.33 (1.14)	0.01	–0.132 (–0.428 to 0.163)	.38
Perceived trustworthiness (1-7), mean (SD)	5.39 (0.70)	5.20 (0.61)	–0.19	–0.199 (–0.382 to −0.016)	.03
Usability/readability (1-7), mean (SD)	5.40 (0.60)	5.39 (0.66)	–0.01	–0.069 (–0.254 to 0.115)	.46
Cognitive load (0-10), mean (SD)	3.96 (1.60)	3.56 (1.44)	–0.40	–0.346 (–0.762 to 0.071)	.10

a UI: user interface.

b Unadjusted difference was calculated as the Safety UI bundle arm mean minus the Baseline UI arm mean, in the original units of each outcome.

c Adjusted estimates controlled for age, gender, education, prior AI chat exposure, digital literacy, technology anxiety, and assistance mode.

### Heterogeneity and Sensitivity Analyses

The condition-by-digital literacy interaction for verification intention was near zero (β=−0.005, 95% CI –0.220 to 0.210; *P*=.96), suggesting that the Safety UI bundle effect on verification intention was broadly similar across the observed literacy range. To evaluate whether retaining participants who required family or interviewer assistance materially changed the observed intervention effect, we repeated the analysis among participants who completed the survey independently (n=134). The pattern remained consistent, with mean verification intention 4.50 (SD 0.58) in the Baseline UI arm and 4.70 (SD 0.66) in the Safety UI bundle arm. In the covariate-adjusted model restricted to independent completers, assignment to the Safety UI bundle was associated with higher verification intention (adjusted β=0.218, 95% CI −0.025 to 0.461; *P*=.08). The estimate was directionally consistent with the primary analysis, although the CI included zero.

Sensitivity analyses using complete-case data were identical to the main analytic sample because missingness did not exceed the prespecified threshold. Gender and education showed the most visible between-arm differences; because both were prespecified covariates in the primary adjusted models, these imbalances were already accounted for, and no separate post hoc adjustment was required.

## Discussion

### Principal Findings

The safety UI bundle was associated with higher verification intention on a 7-point scale, with an adjusted difference of about 0.29 and a moderate standardized effect. Reliance intention did not increase and showed a small, nonsignificant decrease, indicating that the bundle shifted users toward more verification without encouraging greater direct reliance.

### Comparison With Current Literature

The observed increase in verification intention with the safety UI bundle aligns with a growing body of human-centered AI and automation trust research demonstrating that interface cues, such as uncertainty indicators, source displays, or explicit verification prompts, can meaningfully shift user behavior toward more critical engagement and reduce blind reliance on AI outputs [3,4,21]. Prior studies have shown that while explanations and transparency features often increase users’ trust in AI systems, they do not always translate into greater verification or scrutiny of outputs [14-16]. In contrast, direct verification nudges, such as prompts to check sources or warnings about potential errors, have been found to be more effective at encouraging users to verify information rather than simply increasing their confidence in the system [3,17,18]. The effect size observed here is comparable to those reported for other UI interventions aimed at mitigating misinformation or promoting critical evaluation, such as warning labels and uncertainty communication strategies [17,22]. Notably, such interventions can shift intentions even when the underlying content quality remains unchanged, underscoring the value of interface-level design for safety [3,22,23].

This pattern supports trust calibration frameworks emphasizing that safe adoption of AI is not about simply increasing or decreasing trust but about aligning user reliance with system uncertainty and capability [4,24-26]. Overreliance, sometimes termed “automation bias,” is a well-documented risk when systems appear authoritative or present information without cues about limitations [24,27,28]. By explicitly framing uncertainty and encouraging cross-checking, the safety UI bundle likely counteracted authority cues that can lead to excessive deference to AI recommendations [17,19,29]. Similar effects have been observed in clinical decision support and navigation aids, where calibrated trust interventions reduced overreliance without undermining appropriate use [14,29,30]. The present findings extend this literature by demonstrating that such calibration can be achieved through simple UI modifications in a vignette setting with older adults, a group often considered at higher risk for overtrust due to lower digital literacy or unfamiliarity with automated systems [27,31].

The modest increase in behavioral verification proxies echoes prior research documenting gaps between stated intentions (self-report) and observable behaviors in digital environments [3,32,33]. Behavioral measures in survey-based experiments often yield smaller effects and greater variance than self-reported scales, particularly when the behavior is low-cost (eg, clicking to expand a source) and optional [23,32,33]. Stronger behavioral endpoints, such as actual web searching, time spent reviewing sources, or correctness-based incentives, tend to produce more robust effects but are less feasible in vignette studies [23,32]. Nonetheless, prior work suggests that even modest, noisy behavioral uplifts can support the directionality of intervention effects, especially when triangulated with self-report and manipulation checks [32,33]. The findings highlight both the promise and limitations of using lightweight behavioral proxies in survey experiments and underscore the need for richer behavioral tracking in future research [32,33].

This result contrasts with some studies on warning labels and uncertainty displays that report reduced comprehension or increased confusion, particularly among older adults or users with lower literacy [27,34,35]. In other cases, disclaimers or uncertainty cues have distracted users or reduced perceived clarity of information [34,35]. The present design may have avoided the pitfalls by employing large-font screenshots, a stable layout, and concise cues placed below the answer, features known to support comprehension and minimize cognitive load for older users [27,31]. While prior work sometimes finds that added UI elements increase cognitive load or reduce clarity, the nonworsening comprehension observed here suggests that conservative answer structure and scenario simplicity can help preserve understanding even when safety cues are introduced [27,31,34].

The observed reduction in perceived trustworthiness is consistent with research showing that explicit uncertainty communication can lower perceived competence or reliability of AI systems, even as it improves trust calibration and reduces overreliance [4,17,19]. Citation displays alone often increase perceived credibility and may inadvertently encourage overtrust; however, bundling citations with an uncertainty nudge appears to temper this effect by signaling to users that outputs should be verified rather than accepted at face value [16,17,19]. This aligns with literature distinguishing “trust” from “appropriate trust,” arguing that a small reduction in perceived trustworthiness may be acceptable, or even desirable, if it prevents overreliance on potentially fallible systems [4,19,29]. Similar trade-offs have been reported in studies of warning labels, misinformation interventions, and human-automation warning systems where increased vigilance comes at the cost of slightly diminished confidence in system outputs [17,22].

The findings are encouraging given gerontechnology and accessibility research emphasizing that additional UI elements can burden older adults, especially on small screens or in chat interfaces [27,31,34]. Prior studies have found that added explanations or complex provenance panels can reduce usability scores among older users or those with limited digital literacy [4,16]. The cues used here were intentionally short, visually distinct, and placed in predictable locations, a design approach similar to minimalistic “just-in-time” warnings shown to preserve usability while enhancing safety in HCI research [27,36]. If previous work has argued that safety cues harm user experience, the null usability differences suggest that careful cue design can maintain user satisfaction even as verification orientation is improved [27,36].

High rates of cue recognition bolster confidence in the internal validity of the experimental manipulation. Prior UI cue experiments have sometimes endured manipulation failure, where participants do not notice disclaimers or misunderstand uncertainty indicators, undermining inference about intervention effects [3,19]. Salience and comprehensibility of cues are especially critical for older populations who may miss subtle interface changes due to sensory or cognitive constraints [27,31]. The rates of cue recognition observed here appear comparable to those reported for effective labels and warnings in digital interfaces; design choices such as clear visual separation and concise language likely contributed to this salience without increasing burden [27,36].

While some studies suggest low-literacy users benefit more from explicit guidance or verification prompts [34,37], no meaningful moderation was observed here. Possible explanations include a restricted range of digital literacy within the sample, assistance reducing effective literacy gaps during completion, or the simplicity of the cue making it broadly effective across subgroups [34,37]. In contrast to literature showing strong moderation by education or eHealth literacy on intervention effects [37], the results suggest that well-designed safety UI bundles may be broadly applicable. Nevertheless, targeted tests in more diverse or lower-literacy samples remain warranted to confirm generalizability [37].

### Strengths, Limitations, and Implications

This study used randomized assignment to estimate the effect of a practical safety UI bundle on verification orientation in a generative AI chat interface, while holding prompt and answer content constant. The design targets older Chinese adults, a population with high potential benefit from generative AI assistance but potential vulnerability to automation bias, low digital literacy, and difficulty evaluating AI-generated information.

Several limitations should be considered. Outcomes were based primarily on self-reported intentions within a vignette screenshot setting rather than observed real-world behavior in deployed systems. Static screenshots may understate the cognitive demands of real-time generative AI interaction, where users formulate follow-up questions, interpret changing responses, and decide whether to verify under time pressure. The behavioral proxy was minimal and may not capture substantive verification actions such as checking external sources or consulting professionals. Because assisted completion was permitted for accessibility, some participants with visual barriers may have received the source labels and the uncertainty/verification nudge through verbatim oral reading by a family member or interviewer. For these participants, the intervention may have functioned partly as a verbally mediated warning rather than a purely visual UI nudge. Although assistance mode was recorded, adjusted for, and examined in sensitivity analyses, the study cannot fully isolate visual salience from orally mediated cue exposure among assisted participants.

The study tested a bundle rather than individual safety cues, so it cannot determine whether source labels, uncertainty messaging, or verification nudges drove the observed effects. The generic source chips were useful for avoiding false provenance but may have reduced ecological realism by removing the cognitive burden of evaluating real citations. Because the verification nudge explicitly recommended checking authoritative sources and consulting professionals, verification-intention items may have been susceptible to demand characteristics or social desirability bias. The 2 scenarios were intentionally low risk and conservatively written, which may have produced high comprehension scores and limited the ability to detect comprehension trade-offs during more complex, ambiguous, or error-containing AI responses. The answers did not contain false or hallucinated information, so reduced reliance may partly reflect undertrust of accurate content rather than trust calibration in the strict sense. The cross-sectional design cannot assess habituation, banner blindness, or long-term use. Assistance during completion may have altered subjective cognitive load or comprehension, and on-site staff were not formally blinded after assignment, creating a potential source of interviewer bias. Residual confounding from baseline imbalances, including education and gender, may remain despite covariate adjustment. Finally, the sample size was selected for the continuous primary outcome and was likely underpowered for the binary expansion outcome. Additionally, because several primary or supportive outcomes were tested and no formal alpha adjustment was applied, the possibility of inflated Type I error should be considered.

The findings suggest that simple, implementable safety cues may shift older users toward more verification without degrading comprehension or perceived usability, although perceived trustworthiness may decrease modestly. Design and development teams can treat verification nudges and transparent framing of uncertainty as promising directions for further testing, while recognizing that effects may depend on wording, timing, interface prominence, user characteristics, and whether real citations or interactive system behavior are present.

### Future Work

Future work should test interactive generative AI systems, real verification behavior outside the survey environment, repeated exposure over time, and factorial designs that disentangle source labels, uncertainty language, and verification prompts. Studies should also compare generic labels with real citations to determine whether added ecological realism changes cognitive load, comprehension, and verification behavior.

### Conclusions

In this randomized static-vignette survey, a safety-oriented UI bundle in a generative AI chat interface was associated with higher verification intention among older Chinese adults, while reliance intention, comprehension, and usability/readability remained largely unchanged. The trust calibration index moved in a direction consistent with lower overreliance risk, but interpretation should remain cautious because the study did not include incorrect AI outputs and was not designed to isolate individual UI components.

## Supplementary material

10.2196/94140 Multimedia Appendix 1 Stimulus screenshots of the baseline and safety user interface (UI) conditions.

## Abbreviations

HCI: human-computer interaction
OR: odds ratio
UI: user interface
