# Supplementary material for: Effects of a Safety User Interface Bundle on Verification Intentions in Generative AI Chat Use Among Older Chinese Adults: Randomized Vignette Survey
Source: J Med Internet Res. 2026 Aug 14;28:e94140. doi: 10.2196/94140 (PMC13475784; doi:10.2196/94140)
Supplement: Supplementary file 1 [file jmir-v28-e94140-s001.pdf]

Multimedia Appendix S1. Stimulus screenshots of the baseline and safety UI conditions

Static mobile chat-interface screenshots shown to participants. Within each scenario, the baseline and safety UI versions used identical user prompts and identical AI answer text; the safety UI bundle added two elements below the answer, a generic source-label cue (a“来源” [source] label with neutral, non-clickable source chips and an expandable-source note) and an uncertainty-and-verification nudge panel. Interface layout, typography, spacing, icons, and answer length were held constant across conditions. Interface header (all screens): 智能助手 (“Smart Assistant”). Chinese text is verbatim; English translations are provided in italics.

Figure S1A. Baseline UI, Scenario 1 (everyday service/travel decision)

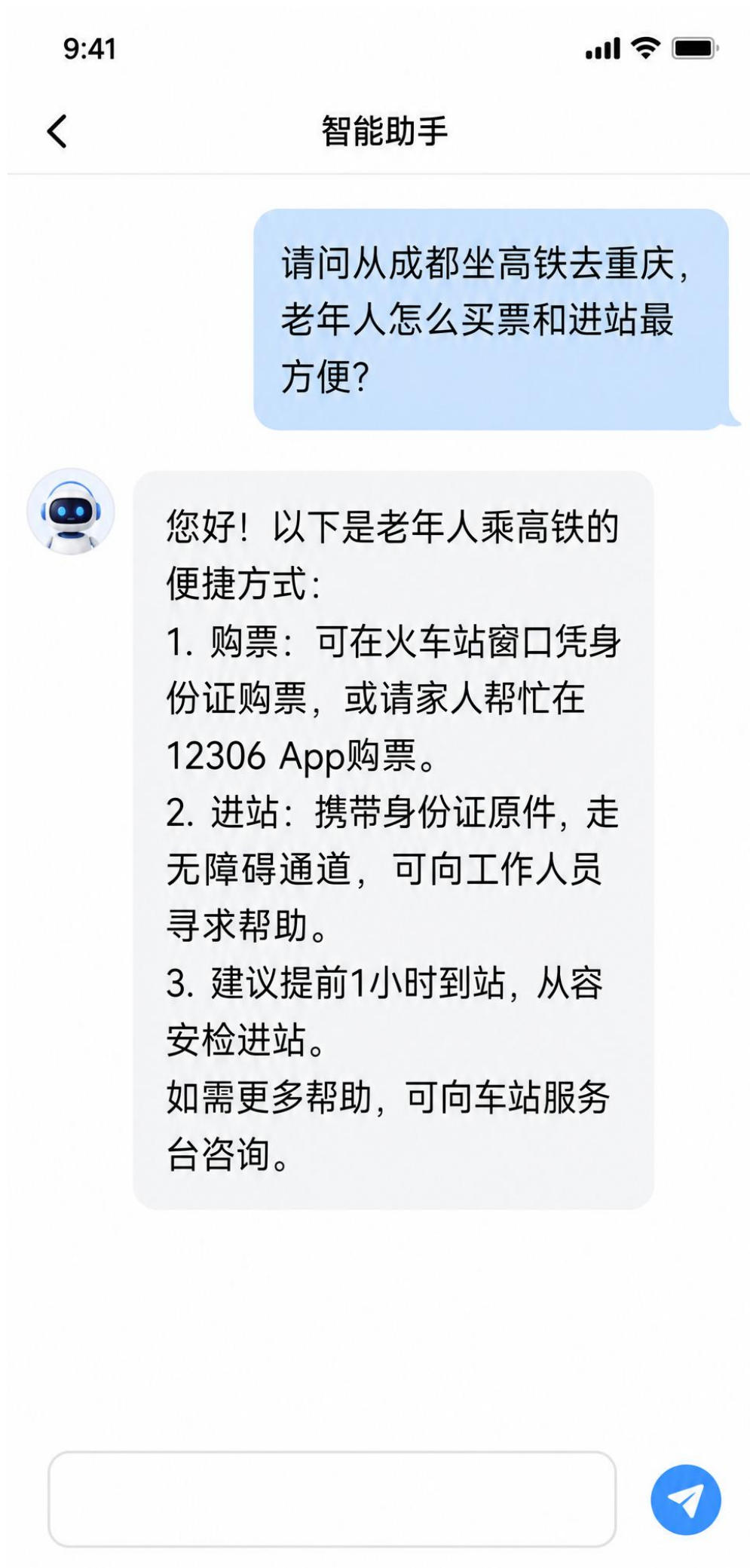

On-screen text (verbatim, with English translation):

User message: 请问从成都坐高铁去重庆，老年人怎么买票和进站最方便？

How can an older person most conveniently buy tickets and enter the station when taking the high-speed train from Chengdu to Chongqing?

AI assistant answer:

您好！以下是老年人乘高铁的便捷方式：

Hello! Here are convenient ways for older adults to travel by high-speed rail:

1. 购票：可在火车站窗口凭身份证购票，或请家人帮忙在 12306 App 购票。

   1. Tickets: buy them at the station counter with your ID card, or ask a family member to help purchase them on the 12306 App.

2. 进站：携带身份证原件，走无障碍通道，可向工作人员寻求帮助。

   2. Entering the station: bring your original ID card, use the accessible passage, and you may ask staff for help.

3. 建议提前 1 小时到站，从容安检进站。

   3. It is advisable to arrive one hour early so you can pass security unhurried.

如需更多帮助，可向车站服务台咨询。

If you need more help, you can ask at the station service desk.

Below the answer: no source-label cue and no uncertainty/verification nudge (baseline condition).

Figure S1B. Safety UI bundle, Scenario 1 (everyday service/travel decision)

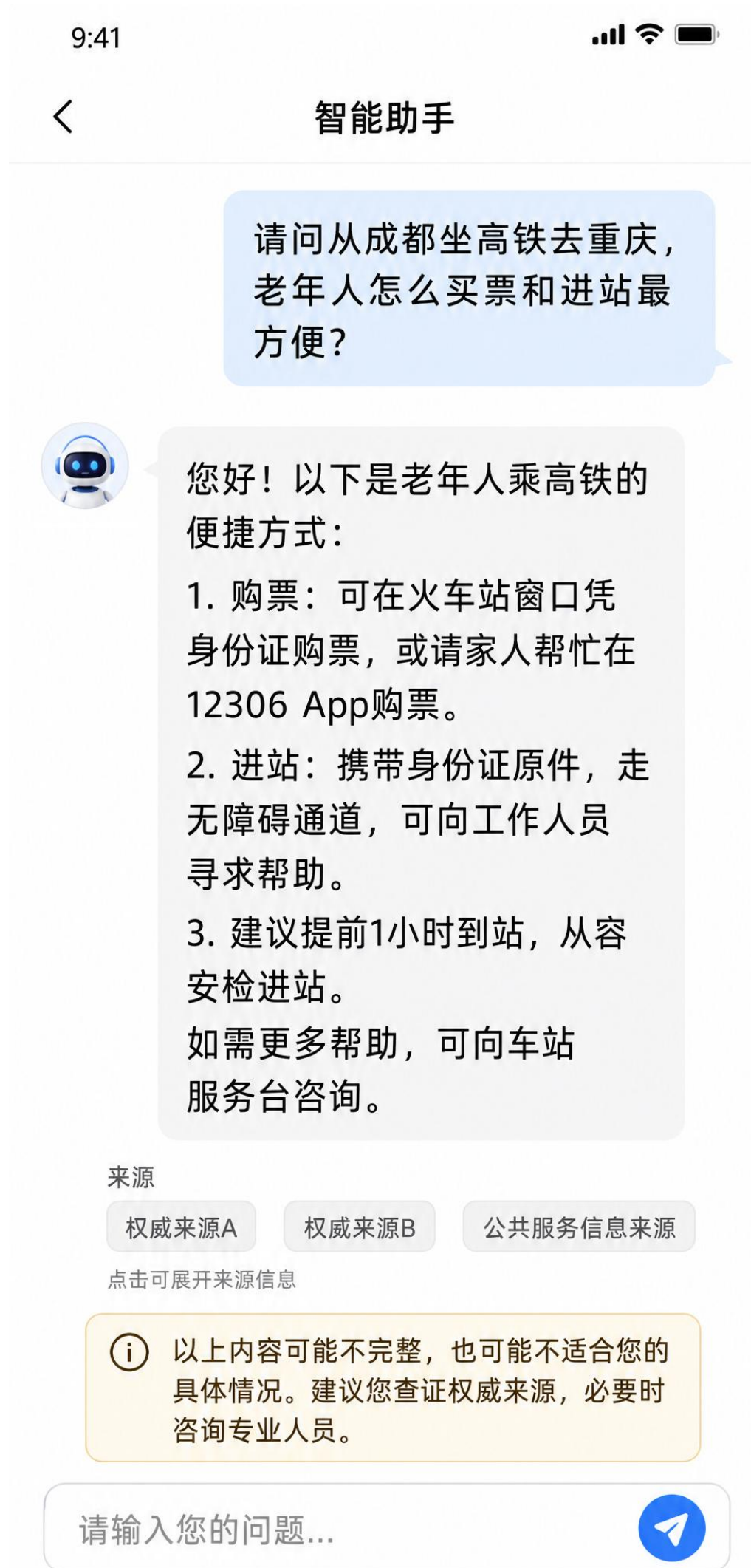

On-screen text (verbatim, with English translation):

User message: 请问从成都坐高铁去重庆，老年人怎么买票和进站最方便？

How can an older person most conveniently buy tickets and enter the station when taking the high-speed train from Chengdu to Chongqing?

AI assistant answer:

您好！以下是老年人乘高铁的便捷方式：

Hello! Here are convenient ways for older adults to travel by high-speed rail:

1. 购票：可在火车站窗口凭身份证购票，或请家人帮忙在 12306 App 购票。

1. Tickets: buy them at the station counter with your ID card, or ask a family member to help purchase them on the 12306 App.

2. 进站：携带身份证原件，走无障碍通道，可向工作人员寻求帮助。

2. Entering the station: bring your original ID card, use the accessible passage, and you may ask staff for help.

3. 建议提前 1 小时到站，从容安检进站。

3. It is advisable to arrive one hour early so you can pass security unhurried.

如需更多帮助，可向车站服务台咨询。

If you need more help, you can ask at the station service desk.

Source-label cue, chips: 权威来源 A ｜ 权威来源 B ｜ 公共服务信息来源

Authoritative Source A | Authoritative Source B | Public Service Information Source

Expandable-source note: 点击可展开来源信息

Tap to expand source information

Uncertainty & verification nudge: 以上内容可能不完整，也可能不适合您的具体情况。建议您查证权威来源，必要时咨询专业人员。

The above may be incomplete or may not suit your specific situation. We suggest checking authoritative sources and consulting a professional when appropriate.

Figure S1C. Baseline UI, Scenario 2 (general wellbeing: sleep/fatigue)

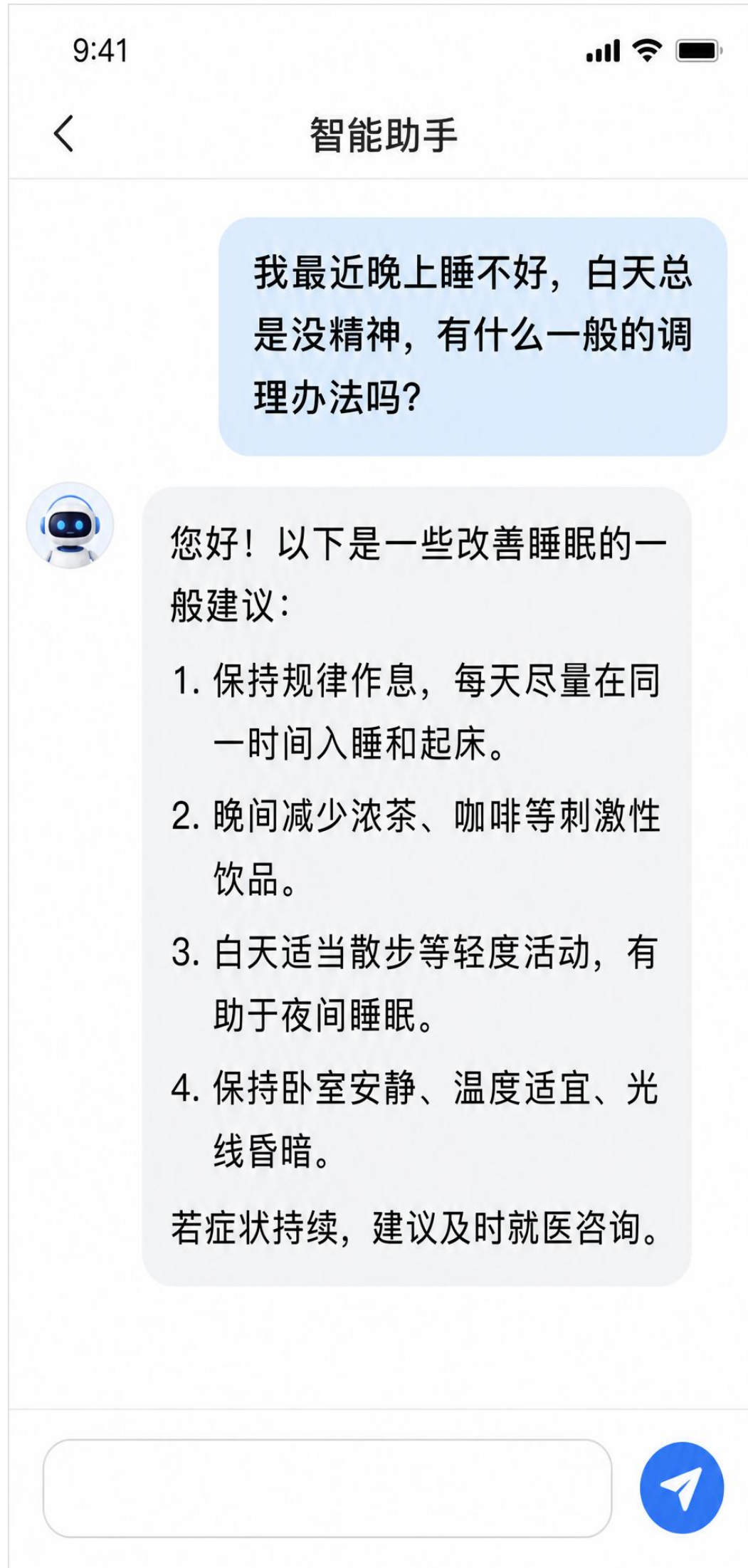

On-screen text (verbatim, with English translation):

User message: 我最近晚上睡不好，白天总是没精神，有什么一般的调理办法吗？

I haven't been sleeping well at night and feel tired during the day; are there general ways to improve this?

AI assistant answer:

您好！以下是一些改善睡眠的一般建议：

Hello! Here are some general suggestions for improving sleep:

1. 保持规律作息，每天尽量在同一时间入睡和起床。

1. Keep a regular routine; try to go to sleep and wake up at the same time each day.

2. 晚间减少浓茶、咖啡等刺激性饮品。

2. Reduce stimulating drinks such as strong tea and coffee in the evening.

3. 白天适当散步等轻度活动，有助于夜间睡眠。

3. Light daytime activity such as walking can help nighttime sleep.

4. 保持卧室安静、温度适宜、光线昏暗。

4. Keep the bedroom quiet, at a comfortable temperature, and dimly lit.

若症状持续，建议及时就医咨询。

If symptoms persist, seek medical advice promptly.

Below the answer: no source-label cue and no uncertainty/verification nudge (baseline condition).

Figure S1D. Safety UI bundle, Scenario 2 (general wellbeing: sleep/fatigue)

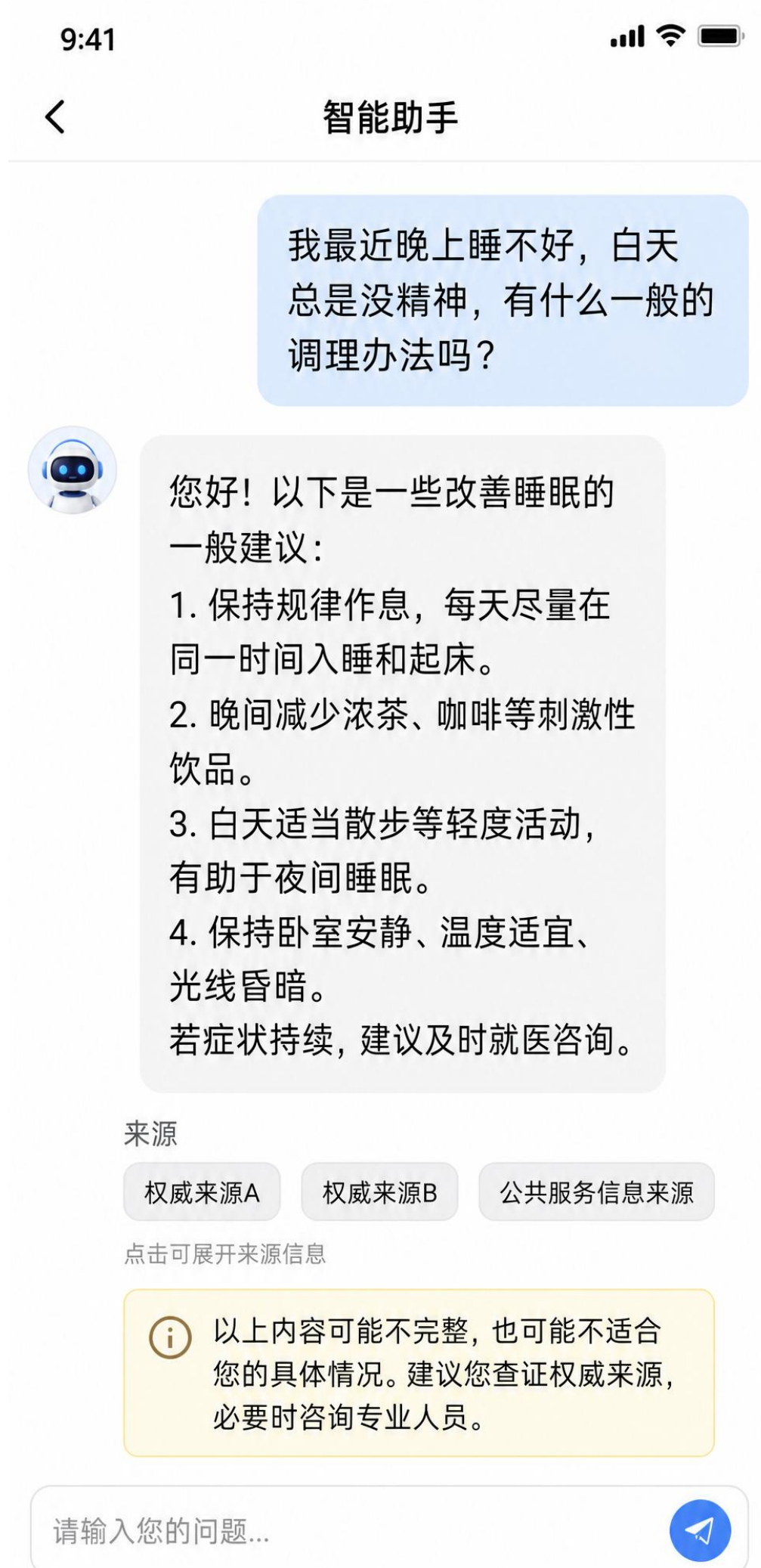

On-screen text (verbatim, with English translation):

User message: 我最近晚上睡不好，白天总是没精神，有什么一般的调理办法吗？

I haven't been sleeping well at night and feel tired during the day; are there general ways to improve this?

AI assistant answer:

您好！以下是一些改善睡眠的一般建议：

Hello! Here are some general suggestions for improving sleep:

1. 保持规律作息，每天尽量在同一时间入睡和起床。

1. Keep a regular routine; try to go to sleep and wake up at the same time each day.

2. 晚间减少浓茶、咖啡等刺激性饮品。

2. Reduce stimulating drinks such as strong tea and coffee in the evening.

3. 白天适当散步等轻度活动，有助于夜间睡眠。

3. Light daytime activity such as walking can help nighttime sleep.

4. 保持卧室安静、温度适宜、光线昏暗。

4. Keep the bedroom quiet, at a comfortable temperature, and dimly lit.

若症状持续，建议及时就医咨询。

If symptoms persist, seek medical advice promptly.

Source-label cue, chips: 权威来源 A ｜ 权威来源 B ｜ 公共服务信息来源

Authoritative Source A | Authoritative Source B | Public Service Information Source

Expandable-source note: 点击可展开来源信息

Tap to expand source information

Uncertainty & verification nudge: 以上内容可能不完整，也可能不适合您的具体情况。建议您查证权威来源，必要时咨询专业人员。

The above may be incomplete or may not suit your specific situation. We suggest checking authoritative sources and consulting a professional when appropriate.
